# The Role of Nuclear Medicine in the Diagnostic Work-Up of Athletes: An Essential Guide for the Sports Cardiologist

**DOI:** 10.3390/jcdd11100306

**Published:** 2024-10-03

**Authors:** Alessandro Zorzi, Sergei Bondarev, Francesca Graziano, Annagrazia Cecere, Andrea Giordani, Luka Turk, Domenico Corrado, Pietro Zucchetta, Diego Cecchin

**Affiliations:** 1 Department of Cardiac, Thoracic, Vascular Sciences and Public Health, University of Padova, Via Giustiniani 2, 35128 Padova, Italy; sergei.bondarev@unipd.it (S.B.); francesca.graziano@unipd.it (F.G.); annagrazia.cecere@unipd.it (A.C.); andrea.giordani@unipd.it (A.G.); domenico.corrado@unipd.it (D.C.); 2 Unit of Nuclear Medicine, Department of Medicine, Università University Hospital of Padova, 35128 Padua, Italy; luca.turk@studenti.unipd.it (L.T.); pietro.zucchetta@aopd.veneto.it (P.Z.); diego.cecchin@unipd.it (D.C.)

**Keywords:** arrhythmogenic cardiomyopathy, coronary artery disease, congenital coronary anomalies, myocardial bridging, sports cardiology, sudden cardiac death, ventricular arrhythmias

## Abstract

Athletes with heart disease are at increased risk of malignant ventricular arrhythmias and sudden cardiac death compared to their sedentary counterparts. When athletes have symptoms or abnormal findings at preparticipation screenings, a precise diagnosis by differentiating physiological features of the athlete’s heart from pathological signs of cardiac disease is as important as it is challenging. While traditional imaging methods such as echocardiography, cardiac magnetic resonance, and computed tomography are commonly employed, nuclear medicine offers unique advantages, especially in scenarios requiring stress-based functional evaluation. This article reviews the use of nuclear medicine techniques in the diagnostic work-up of athletes with suspected cardiac diseases by highlighting their ability to investigate myocardial perfusion, metabolism, and innervation. The article discusses the application of single photon emission computed tomography (SPECT) and positron emission tomography (PET) using radiotracers such as [99mTc]MIBI, [99mTc]HDP, [18F]FDG, and [123I]MIBG. Several clinical scenarios are explored, including athletes with coronary atherosclerosis, congenital coronary anomalies, ventricular arrhythmias, and non-ischemic myocardial scars. Radiation concerns are addressed, highlighting that modern SPECT and PET equipment significantly reduces radiation doses, making these techniques safer for young athletes. We conclude that, despite being underutilized, nuclear medicine provides unique opportunities for accurate diagnosis and effective management of cardiac diseases in athletes.

## 1. Introduction

Exercise can be a potential trigger of ventricular arrhythmias (VA) in athletes with underlying heart disease [1,2,3,4]. Distinguishing between physiological adaptations of the athlete’s heart and pathological cardiac conditions presents significant challenges, complicating the diagnosis and risk stratification of athletes with suspected cardiac diseases [5]. Cardiovascular imaging is commonly used in the diagnostic work-up and risk stratification of athletes. Specifically, echocardiography, and more recently, contrast enhanced magnetic resonance imaging (MRI) and coronary computed tomography (CCTA), are tools employed in clinical practice [6,7,8]. Conversely, nuclear medicine techniques, despite being widely used in clinical cardiology, are rarely considered for application in the diagnostic work-up of athletes with suspected heart disease. However, there are specific areas of application where nuclear medicine techniques could be particularly useful for athletes.

This article aims to provide an essential guide for sports cardiologists, particularly those who may not be familiar with the use of nuclear medicine techniques in their clinical practice. Our objective is to introduce the basic principles and highlight specific clinical scenarios where these methods can aid in decision-making, thereby enriching the cardiologist’s diagnostic toolbox when evaluating athletes with suspected cardiac conditions.

## 2. Causes of Sudden Cardiac Death in Athletes and Potential Role of Nuclear Medicine in Their Diagnostic Work-Up

Despite the well-known benefits of regular physical exercise, intense and continuous training can trigger VA and fibrillation in athletes with an underlying heart disease. This explains the higher incidence of sudden cardiac arrest among athletes compared to nonathletes [1,2]. The causes of sudden cardiac death (SCD) in athletes vary depending on age. In younger athletes, under 35–40 years, a variety of conditions, including congenital coronary artery anomalies, inherited conditions such as hypertrophic cardiomyopathy, ion channel diseases, and other diseases can cause ventricular arrhythmias. Conversely, in older athletes, most cases of SCD are caused by complications of acute coronary artery disease [2].

The mechanism by which physical exercise can cause VA is not completely understood, but a variety of mechanisms have been hypothesized. In particular, it is known that exercise triggers a cascade of reactions including catecholamine activation, oxidative stress, and suppression of natural antioxidant systems, leading to inflammation, disruption of macroerg generation, and protein synthesis in cardiomyocytes. Ultimately, these changes affect membrane function, impacting the transport and accumulation of potassium, magnesium, sodium, and calcium ions. This can lead to electrical instability and a propensity for VA [9,10,11].

When heart disease is suspected in an athlete, a comprehensive diagnostic work-up is crucial. The first step is to differentiate between normal heart rate adaptations to exercise and true cardiac pathology. Secondly, once a cardiac disease is identified, the risk stratification of the condition is necessary, particularly in assessing the propensity for VA during exercise and determining the athlete’s eligibility to continue participating in competitive sports.

Nuclear medicine can play a vital role in these scenarios, offering distinctive capabilities that other diagnostic modalities typically do not possess [12]. Specifically, certain radiopharmaceuticals can be administered during exercise testing, allowing for evaluating what happens during true exercise rather than just pharmacological testing. This approach more accurately replicates the physical exertion an athlete undergoes during daily activities. Additionally, radiopharmaceuticals can evaluate physiological and pathophysiological aspects of cardiac muscles that other diagnostic methods cannot. For these reasons, nuclear medicine can be particularly useful in the diagnostic work-up of athletes with suspected cardiac disease. It offers unique insights and capabilities that enhance the accuracy of diagnosis and risk assessment.

## 3. Nuclear Medicine Techniques

**Figure 1:**
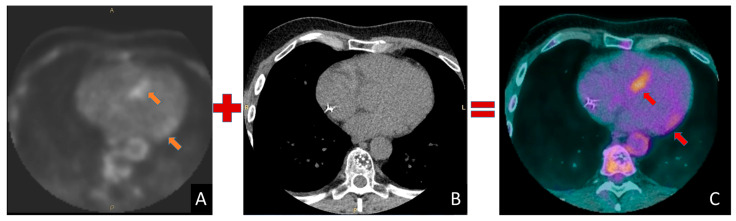
Representative example of a PET-TC merged image. (**A**) PET shows increased FDG uptake in the basal septum and basal lateral wall of the left ventricle (arrows). (**B**) chest CT image, (**C**) merged PET and TC image that allows better localization of the areas with increased uptake (arrows).

The basic principle underlying nuclear medicine techniques is to “attach” a radioactive isotope to a “drug”, which can be something as simple as glucose or sodium, receptor-targeting drugs that bind to specific receptors, or pharmaceuticals that are taken up within specific cellular organelles, such as mitochondria. The purpose of labelling these molecules with a radioactive isotope is to study their distribution within the body. Once the radiopharmaceutical is taken up or accumulated in a specific region of the body, it emits radiations (gamma rays or positrons) that are detected (with high sensitivity as compared to other techniques) by a specialized detector outside the patient. The software integrated into the scanner then provides a three-dimensional reconstruction of the radioactive emission source. This allows for the study of the distribution and concentration of the radiopharmaceutical in various tissues and organs [13]. Nuclear medicine instrumentations are now invariably coupled with a CT or MRI scanner to allow multimodal acquisitions. TC is primarily used for attenuation correction, i.e., the process of compensating for the absorption of gamma rays by body tissues, which can otherwise distort the images. In addition, both CT and MRI provide detailed anatomical images that can be fused with functional images to better localize pathological findings. MRI, when coupled with PET in PET/MRI, offers superior soft-tissue contrast and the ability to assess both functional and anatomical abnormalities without additional ionizing radiation (Figure 1).

### 3.1. SPECT versus PET

Single photon emission computed tomography (SPECT) uses specialized collimators and detectors constituted of scintillation crystals or semiconductor materials such as the CZT (cadmium zinc telluride) technology. Detectors convert gamma rays (from gamma emitting radiopharmaceuticals) into electrical energy [14].

Positron emission tomography (PET) operates on similar principles but detects pairs of oppositely moving gamma rays derived from the annihilation of positrons (β+). The primary difference between PET and SPECT (apart from the different radiopharmaceuticals available in the two modalities) lies in the emission of photon pairs in PET compared to single gamma photons in SPECT. Due to this and a number of other technical issues that go beyond the scope of this paper, PET usually achieves better contrast and spatial resolution and in a shorter time compared to SPECT [15].

### 3.2. Radiotracers

The most commonly used radiotracers for SPECT are based on Technetium-99m (e.g., [99mTc]MIBI, [99mTc]-tetrafosmin). Sodium pertechnetate 99mTc is obtained from Molybdenum-99Mo using a generator and combined with sestamibi (MIBI) or tetrofosmin, which penetrate cardiomyocytes. Both MIBI and tetrofosmin are taken up by the mitochondria of cardiomyocytes in proportion to metabolic activity (higher uptake during stress than at rest, and reduced uptake in fibrotic tissue compared to viable myocardium) and myocardial perfusion (reduced uptake in hypoperfused areas compared to normally perfused areas). Since these radiopharmaceuticals remain in the mitochondria for several hours, continuing to emit radioactivity, it is possible to acquire SPECT images at various times after the injection of the radiopharmaceutical. These radiotracers are not significantly washed out (low redistribution) of the myocardium during the exams and do not require blood flow correction. The primary application of myocardial SPECT with 99mTc-labeled MIBI or tetrofosmin is the study of myocardial perfusion at rest and after physical or pharmacological stress [16]. 99Tc-pyrophosphate binds to extracellular calcium, aiding in the diagnosis of acute myocardial ischemia and cardiac amyloidosis [17]. [201Tl]-chloride, an analog of potassium, enters cells via Na+/K+-ATPASE and electric potential gradients, redistributing in the myocardium and persisting in ischemic zones to assess myocardial viability [18]; however, it is used less often because of its unfavorable emitting characteristics. Another tracer, [123I] MIBG, used in cardiac failure and in the differential diagnosis of some neurodegenerative forms, accumulates in presynaptic adrenergic membranes, reflecting changes in adrenergic activity [19].

Commonly used radiotracers for PET include 82Rb-chloride and 13N ammonium. 82Rb-chloride, a potassium analog, enters cardiomyocytes via Na+/K+- ATPase and is produced by an 82Sr/82Rb generator. Another tracer, 13N-ammonium, diffuses through membranes and is enzymatically converted to glutamine in cardiomyocytes. However, 13N’s short half-life (9.9 min) necessitates on-site production, limiting its use to centers equipped with a cyclotron. Another example is [18F]flurpiridaz, a novel PET myocardial perfusion imaging tracer. It inhibits NADH ubiquinone oxidoreductase in the mitochondrial complex-1 electron transport chain, providing high-quality images and optimal resolution. Clinical studies have shown that 18F flurpyridaz has superior diagnostic performance compared to traditional tracers such as 82Rb chloride and 13N-ammonium, and has a longer half-life that makes it suitable for widespread use, including in exercise stress tests [20] (Table 1).

### 3.3. Radiation Exposures

Nuclear medicine techniques require the use of radiopharmaceuticals that emit ionizing radiation, and therefore, the risks associated with radiation exposure, similar to other radiological exams, must be considered before prescribing the test. This is particularly important for young athletes and even more so for female athletes, where the risk of radiation-induced adverse events, such as neoplasms, can manifest much later in life. Therefore, it is crucial to always evaluate the risk–benefit ratio of prescribing one of these tests, considering the radiation dose and possible alternatives.

Regarding the radiation dose for a stress-rest myocardial scintigraphy, which is the most commonly used test in nuclear cardiology, the dose patients are exposed to varies significantly depending on the equipment used. With older generation machines, such as traditional SPECT cameras based on sodium iodide crystals doped with thallium, the average radiation dose for a stress-rest test is quite high, reaching up to approximately 14 mSv. However, with the new CZT (cadmium zinc telluride) technology, it is possible to significantly reduce the radiation dose down to values of about 3–5 mSv (for the entire stress + rest procedure). CZT furthermore allows for faster acquisitions (3–5 vs. 20–30 min) and simplified quality control procedures. These dose reductions are possible due to the greater sensitivity and better geometry of CZT detectors, as well as advanced image reconstruction algorithms, which contribute to obtaining high-quality images while using a much lower dose of a radiopharmaceutical. This brings the dose to a level comparable to or even lower than that of a high-quality CCTA scan and does not require the use of iodinated contrast agents [21].

Specific clinical questions can only be investigated by methods that involve the use of ionizing radiation, such as [18F]FDG PET for suspected sarcoidosis. However, for the detection of inducible ischemia, methods such as stress echocardiography could be reasonable alternatives, especially in young patients. It should be noted that pharmacological stress echocardiography cannot accurately reproduce what happens during physical exercise, which is particularly important when assessing specific ischemic substrates, such as coronary anomalies. Physical stress echocardiography using a bicycle ergometer could be considered, keeping in mind that young athletes can reach heart rates well above 150 beats per minute during exertion, which could make the evaluation of small regional wall motion abnormalities less accurate. Moreover, it has been demonstrated that the maximal oxygen consumption in active healthy individuals is significantly lower when using semi-supine or supine-cycle ergometers compared to standard ones [22]. Therefore, when assessing the risk-benefit ratio of subjecting a young athlete to an ionizing radiation examination such as myocardial scintigraphy, the radiation risk must certainly be considered. However, it should also be noted that scintigraphy is a very sensitive method for detecting inducible ischemia and allows for an evaluation during maximal physical stress that is not affected by potential artifacts associated with high heart rates [12].

## 4. Clinical Scenarios of Potential Utility of Nuclear Medicine in Current Sports Cardiology Practice

### 4.1. Athlete with Positive Exercise Testing and Moderate Coronary Stenosis

Coronary atherosclerosis is the primary cause of adverse cardiovascular events and sudden death in athletes over the age of 35–40, particularly in males [1,2]. From the perspective of sports cardiology, the population of master athletes has seen an exponential increase in recent decades. Today, millions of individuals over the age of 40, or even 50–60, participate in competitions requiring intense cardiovascular efforts, such as marathons, cycling, or triathlons [23]. In this population, cardiovascular assessment should primarily focus on excluding underlying coronary artery disease, while also considering the possibility of other conditions that may have developed earlier in life [24].

Recent studies have highlighted the incidence of coronary artery disease (CAD) in master athletes. Although physical activity is traditionally viewed as protective against the development of coronary atherosclerosis, recent evidence suggests a higher incidence of CAD in this population. It has been hypothesized that this may be due to the shear stress on the coronary arteries induced by intense exercise. Furthermore, while initial studies suggested a higher incidence of stable, non-vulnerable plaques, more recent findings indicate an increased incidence of vulnerable soft plaques in athletes [25,26,27].

The best method for screening master athletes for CAD remains to be established. On one hand, sensitive tests such as CCTA are needed, particularly in those with high cardiovascular risk [28]. On the other hand, simpler tests such as the exercise stress test, despite their limitations, are preferable in the context of population screening, where it is impractical to subject millions of individuals to complex and less accessible radiological investigations [23,29,30]. The exercise stress test has reduced sensitivity and specificity, particularly when an athlete presents with a positive test that may not necessarily indicate underlying myocardial ischemia [30]. In such cases, CCTA is often used as the follow-up examination due to its high negative predictive value. According to the 2024 ESC Guidelines for the management of chronic coronary syndromes, in patients with a moderate pre-test likelihood of obstructive coronary artery disease (15–50%), such as a male master athlete with a positive exercise-testing CCTA, is the preferred first-line test due to its high negative predictive value [31]. In other words, if a CCTA is negative for coronary atherosclerosis, the athlete can be considered low risk and allowed to participate [8]. However, the ability of CCTA to discriminate between flow-limiting and non-flow-limiting atherosclerotic lesions, especially in the context of moderate plaques, is limited [30,31,32]. The 2024 ESC Guidelines also acknowledge that the ability of CCTA to discriminate between flow-limiting and non-flow-limiting lesions, particularly in the context of moderate stenosis, is limited [31]—Sequential testing with functional imaging techniques is recommended when the significance of a coronary lesion is uncertain, especially for better assessment of myocardial ischemia. In this scenario, exercise-myocardial scintigraphy is particularly useful because, as highlighted by the ESC guidelines for the management of chronic coronary syndromes and sports cardiology [31,33], functional imaging that utilizes true maximal physical stress is preferred over pharmacological stress whenever possible. This approach provides a more accurate assessment of exercise-induced myocardial ischemia, which is particularly important for athletes, as it better reflects the physiological conditions experienced during competitive or intense physical activity

**Figure 2:**
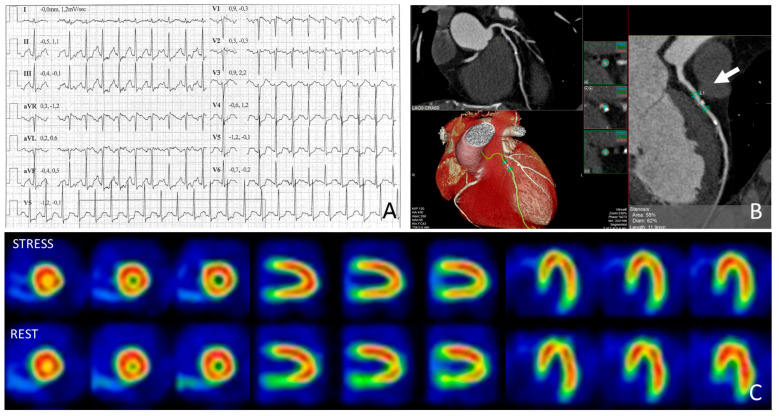
Representative example of an asymptomatic athlete with borderline ST-segment depression performed for preparticipation screening (**A**). Coronary computed tomography revealed moderate stenosis of the left anterior descending artery (**B**). However, stress-rest myocardial SPECT showed no inducible myocardial ischemia (**C**).

For example, consider a 47-year-old male athlete with one or two risk factors (e.g., family history of ischemic heart disease and mild hypertension) who undergoes a screening stress test that is positive for ST-segment depression at peak exercise without symptoms. The athlete is then subjected to CCTA, which reveals a moderate stenosis, leaving the clinician uncertain whether the stress test reflects true inducible ischemia or if it was a false positive due to a non-flow-limiting plaque (Figure 2). The athlete can therefore undergo an inducible ischemia test, and in this context, myocardial scintigraphy is an excellent choice, as it allows him to perform maximal exercise on a bicycle or treadmill before the radiopharmaceutical injection, providing a perfusion image at peak exercise.

### 4.2. Athlete with Positive Exercise Testing and Myocardial Bridging

Anomalies of the origin and path of the coronary arteries are another significant condition to be ascertained in the setting of sports cardiology. These congenital anomalies have the potential to cause a reduction in blood flow to the myocardium, particularly during intense exercise. The potential for ischemia translates into the possibility of inducing exercise-induced VA and thus sudden death. Often, coronary anomalies of origin and course present with subtle or no symptoms, and their presence is once again suggested by a positive stress test performed for screening purposes.

Focusing first on myocardial bridging (MB), it is a relatively common anomaly with prevalences ranging between 26% and 58% in CCTA studies [34,35,36,37]. However, only in a minority of cases does MB result in inducible ischemia, while in other cases it is simply an anatomical variant without clinical relevance. The localization (i.e., more than 2 mm deep in the myocardium vs. superficial) and length (i.e., more than 2.5 cm) of the bridging are key features to predict the functional relevance of MB [34]. In an athlete with a positive stress test, for example due to ST segment depression, as previously mentioned, it is now customary to follow up with CCTA, leveraging its high negative predictive value. It frequently happens that this examination demonstrates the absence of atherosclerotic plaques but reveals the presence of an MB, particularly involving the left anterior descending artery. This scenario is clinically challenging because demonstrating inducible ischemia related to MB is more difficult compared to fixed atherosclerotic plaques.

From a pathophysiological standpoint, coronary blood flow occurs during diastole, whereas MB constricts the coronary lumen particularly during systole. It has been hypothesized that mechanisms causing ischemia due to MB include an altered diastolic relaxation of the coronary lumen, which occurs only when the heart rate increases significantly. In other words, during tachycardia, MB causes delayed restoration of the normal coronary lumen at the beginning of diastole, resulting in reduced flow and potential ischemia. Other mechanisms involve the possibility of a coronary spasm or a Venturi effect steal phenomenon from the intramyocardial segment to its collateral branches. These mechanisms, however, typically occur during intense exercise [34].

For all these reasons, invasive or non-invasive tests using pharmacological stress with vasodilators such as adenosine or dipyridamole are less informative for eliciting ischemic mechanisms related to MB. The use of dobutamine as pharmacological stress, on the other hand, is not standardized in this clinical context and could lead to false positives [30]. It is much more appropriate to subject an athlete to real and truly maximal physical exertion, and the only test in clinical practice that can achieve this is myocardial scintigraphy with physical stress, considering that an echostress on a supine ergometer rarely allows the athlete to reach their maximal oxygen consumption [22]. Accordingly, the 2024 ESC Guidelines for Chronic Coronary Syndromes, the 2020 ESC Guidelines on Sports Cardiology, and the 2020 ESC Guidelines for the Management of Adult Congenital Heart Disease recognize that MB is often an incidental finding but can sometimes lead to exercise-induced myocardial ischemia. In athletes, maximal physical stress testing (that causes both inotropic and chronotropic stimulation) is crucial to assess the functional significance of MB, as it better replicates the physiological conditions of exercise compared to pharmacological stress. This approach is essential in identifying athletes at risk of ischemia or complex arrhythmias during competition, which may limit their participation in high-intensity sports if confirmed [31,33,38].

**Figure 3:**
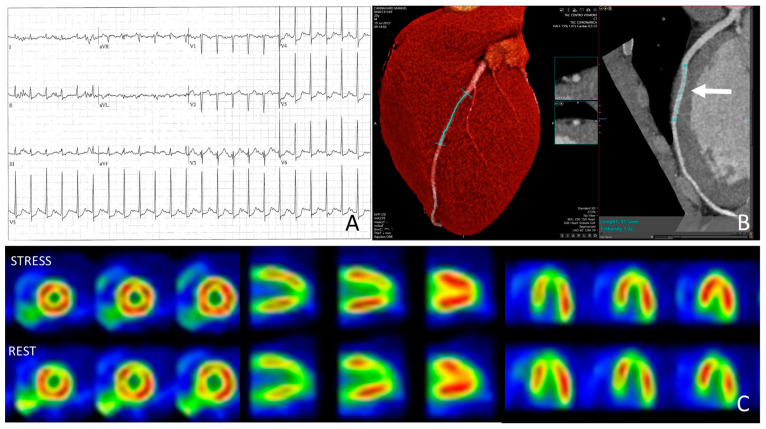
Representative example of an asymptomatic athlete with ST-segment depression performed for preparticipation screening (**A**). Coronary computed tomography revealed myocardial bridging of the left anterior descending artery (**B**). However, stress-rest myocardial SPECT showed no inducible myocardial ischemia (**C**).

Consider, for example, the case of a 40-year-old athlete who is asymptomatic or has reported non-anginal chest pain and undergoes a screening exercise stress test that is positive for inducible ischemia. The athlete then undergoes a CCTA, which is negative for atherosclerotic plaques but reveals a deep and long MB of the left anterior descending artery. To confirm or rule out the presence of inducible ischemia, the athlete is subjected to a stress test with an infusion of MIBI at peak exercise. The athlete reaches 300 watts on the cycle ergometer and a heart rate equal to 102% of the theoretical maximum, with exertion rated as 10 out of 10, thus truly maximal. The SPECT is negative for perfusion defects in the distribution territory of the intramyocardial course, thereby reassuring the clinician about the bystander nature of the detected anomaly (Figure 3).

### 4.3. Athlete with Anomalous Origin of the Coronary Arteries with Interarterial Course

Another challenging scenario in sports cardiology is the management of athletes with anomalous origin of the coronary arteries with interarterial course. Specifically, the origin of the right coronary artery from the left sinus of Valsalva, which is the most common coronary anomaly with an incidence of about 0.3–0.5%, and the origin of the left coronary artery trunk from the right sinus of Valsalva, which is much rarer. There are also even rarer anomalies, such as the origin of a coronary artery from the pulmonary artery, but these usually cause symptoms in childhood and are not considered in this section [35,37,38,39,40,41]. Anomalous origin of coronary arteries from the contralateral sinus of Valsalva can remain asymptomatic throughout life, cause symptoms such as classic effort angina, or more frequently, subtle symptoms such as exercise-induced dyspnea or syncope. They can also cause SCD due to exercise-induced ventricular fibrillation as the first clinical manifestation of the disease. According to several autoptic series, anomalous coronary artery origin is one of the leading causes of SCD in athletes [1,2].

The diagnosis of an anomalous origin of a coronary artery in an athlete can be reached following a diagnostic pathway initiated by a symptom or in an asymptomatic athlete because of a screening test. This could be a stress test or even a basal echocardiogram, as the coronary ostia can be visualized with specific echocardiographic views [7].

In the case of an asymptomatic athlete with an anomalous origin of the coronary artery, the indications for possible surgical treatment and eligibility for competitive sports depend on two morpho-functional characteristics. The first is the potential of the coronary anomaly to cause myocardial ischemia, and the second is the presence of high-risk anatomical features such as slit-like ostium, intramural course, and narrowing of the first coronary segment [40,41].

This anatomical definition necessarily requires an imaging test such as coronary CT angiography (CTA) or coronary angiography. For demonstrating the potential for inducible ischemia, the challenge, similar to that of the intramural course, lies in the fact that traditional inducible ischemia tests involving pharmacological stress with vasodilators show rather low sensitivity. This is because the mechanism of inducible ischemia requires the dynamic “squeezing” of the coronary artery between the aorta and pulmonary artery, which only occurs with significant increases in cardiac output during physical exertion [37]. Coronary angiography with invasive pressure monitoring during dobutamine infusion has been proposed, but again, besides being an invasive test, it lacks validation studies in this context [42].

Therefore, it appears more rational to use a technique that can evaluate potential ischemia induced by real physical exercise. Current guidelines and consensus documents agree that functional imaging with non-pharmacological stress, such as myocardial scintigraphy during physical exertion, is recommended to assess exercise-induced ischemia, which is challenging to detect using pharmacological vasodilators due to the dynamic compression mechanism between the aorta and pulmonary artery during intense physical activity [33,38]. In this context, myocardial scintigraphy is an excellent choice due to the possibility of injecting the radiopharmaceutical at peak exercise and accurately assessing even localized perfusion defects with high sensitivity, which is difficult to achieve with stress echocardiography.

**Figure 4:**
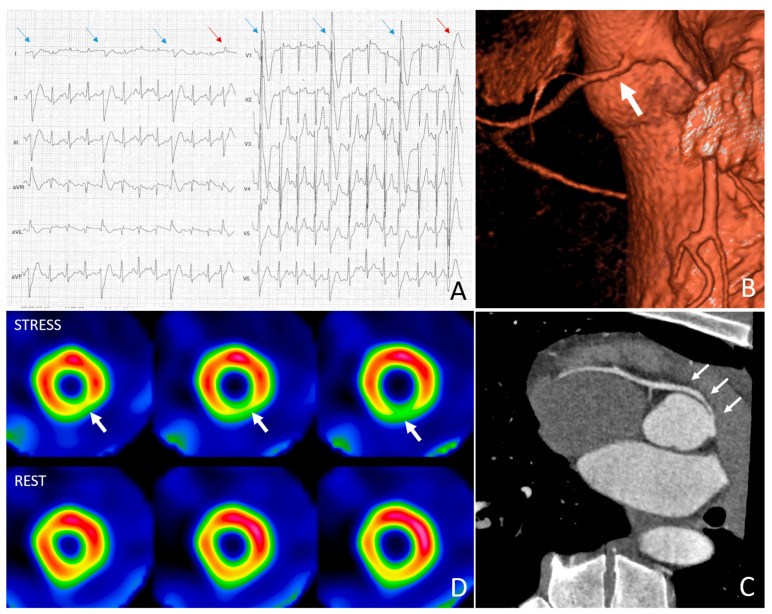
Representative example of an athlete with premature ventricular beats at exercise testing performed for preparticipation screening (**A**). Further examinations revealed a right coronary artery originating from the left Valsalva sinus with slit-like ostium and narrowing of the interatrial course (**B**,**C**). Stress-rest myocardial SPECT revealed reversible ipoperfusion of the infero-lateral left-ventricular wall, tributary to the abnormal coronary artery (**D**).

Consider, for example, the case of a 17-year-old athlete undergoing a medical-sport screening visit during which some exercise-induced ventricular extrasystoles are recorded. Following this finding, an echocardiogram is performed, suggesting the possibility of an anomalous origin of the right coronary artery from the left sinus of Valsalva. Consequently, the athlete undergoes coronary CTA, which confirms the presence of a right coronary artery originating from the left sinus of Valsalva and coursing between the two great vessels to its distribution territory. Anatomically, high-risk features are present because of an intramural first tract with narrowing of the proximal coronary segment. The athlete then undergoes myocardial scintigraphy with physical stress. He reaches an exercise intensity of 350 watts with a peak heart rate of 97% of the theoretical maximum and perceived exertion of 9 out of 10. The SPECT with MIBI is positive for a circumscribed area of inducible ischemia in the inferior and inferolateral wall that, by comparison with images acquired at rest, is considered not justifiable by diaphragmatic attenuation alone. Therefore, the coronary anomaly is considered potentially high-risk, and surgical correction is suggested (Figure 4).

### 4.4. Athlete with Ventricular Arrhythmias and Late Enhancement on Cardiac MRI Suspected for Cardiac Sarcoidosis

Cardiac MRI is a widely used examination in sports cardiology. Indications can vary, but two of the most common are the presence of ECG abnormalities, such as T-wave inversion, or exercise-induced VA, particularly with suspicious characteristics. One of the most challenging scenarios in the clinical context of ECG abnormalities or exercise-induced arrhythmias is the detection of non-ischemic late gadolinium enhancement (LGE). This refers to the presence of gadolinium accumulation in the mid-myocardial and sub-epicardial segments of the left ventricle on T1-weighted post-contrast sequences, suggesting various pathophysiological hypotheses, such as a healed myocarditis, an active inflammatory process, a genetic cardiomyopathy, or, although still speculative, exercise-induced damage [43,44].

Among the diagnostic hypotheses, cardiac sarcoidosis should not be overlooked. Sarcoidosis is a granulomatous disorder that can affect the heart, either in association with other organs, particularly the thoracic-pulmonary lymph nodes, or in an isolated cardiac form. The presence of cardiac sarcoidosis is suggested by certain specific features on cardiac MRI, such as involvement of the interventricular septum. Common MRI findings suggestive of cardiac sarcoidosis include subepicardial and mid-myocardial late LGE in the basal and lateral segments, involvement of the interventricular septum, focal wall thinning, and regional wall motion abnormalities in both ventricles. Additionally, the presence of atrioventricular or intraventricular conduction defects on the ECG reinforces the suspicion [45].

The diagnosis of cardiac sarcoidosis typically requires an endomyocardial biopsy, an invasive test with low sensitivity for this peculiar cardiomyopathy due to the highly focal nature of the granulomatous lesions [46]. An alternative step when faced with a suggestive cardiac MRI in presence of a suggestive clinical scenario is to perform a total-body PET scan with [18F]FDG after a proper diet (PET/MR instead of PET/CT would be preferable in this scenario but it is not so widely available). This scan can detect areas of high glucose metabolism typical of sarcoidosis granulomas, both within the heart and in extracardiac regions [47]. PET is also extremely useful during patients’ follow-up to monitor the response to therapy and detect subclinical relapses, both in the heart or in other organs.

**Figure 5:**
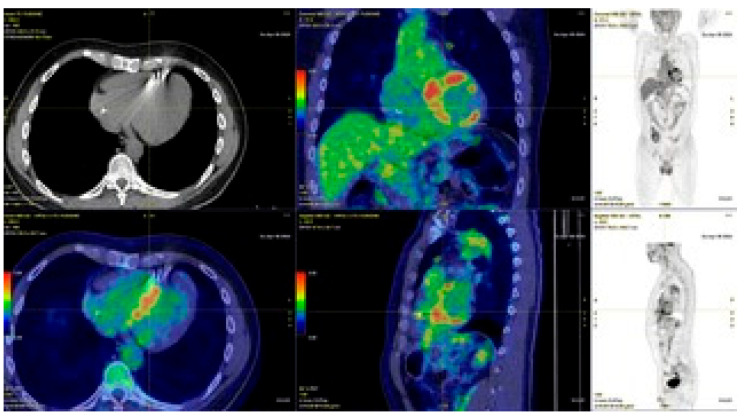
PET-TC with FDG showing disomogeneous intense myocardial uptake, involving the interventricular septum and inferior wall.

Consider the example of a 34-year-old asymptomatic athlete who undergoes preparticipation screening. Resting ECG shows right bundle branch block and polymorphic premature ventricular beats are present on exercise testing. For this reason, he is referred for cardiac MRI, which shows multiple areas of non-ischemic LGE, also involving the interventricular septum. To rule out the possibility of sarcoidosis, the athlete undergoes an FDG-PET scan, which demonstrates pathological tracer accumulation at the myocardial lesions identified on MRI and a peritracheal lymphadenopathy with high glucose metabolism (Figure 5). Thus, the suspicion of sarcoidosis is reinforced, and the patient is subjected to a transbronchial biopsy, confirming the diagnosis by demonstrating typical sarcoidosis granulomas.

### 4.5. Senior Athlete with Unexplained Myocardial Hypertrophy and Suspected Cardiac Amyloidosis

Cardiac amyloidosis is an extracellular infiltrative disease characterized by the deposition of amyloid fibrils within the myocardial interstitial tissue. This results in left ventricular (LV) hypertrophy that is not explained by hemodynamic conditions (i.e., volume overload, arterial hypertension, etc.) and, over time, leads to increased wall stiffness, causing diastolic dysfunction (restrictive diastolic filling pattern) and heart failure with preserved ejection fraction. There are two main types of amyloidosis that can involve the heart. AL amyloidosis is the result of the production of light chains by a plasma cell clone, making it a paraneoplastic complication of hematological cancers, especially multiple myeloma. Transthyretin (aTTR) amyloidosis, on the other hand, can occur in its wild-type form, which is typical of older age, or in its mutated form, which is rarer and often associated with neuropathy [48]. Diagnosing cardiac amyloidosis can be challenging, especially in athletes, where physiological hypertrophy due to intense training can mask the disease. The differential diagnosis between physiological hypertrophy due to training and pathological hypertrophy requires the evaluation of several parameters. Typically, an athlete’s heart exhibits eccentric hypertrophy with balanced dilation of the left and right ventricular chambers and mild wall hypertrophy that generally does not exceed 12–13 mm. Diastolic function is normal, and the atria are at most mildly dilated, proportionally to the dilation of the ventricles [49]. Conversely, in amyloidosis, concentric hypertrophy is observed with increased LV wall thickness but normal volume, non-dilated chambers, and altered diastole, leading to disproportionate left atrial dilation compared to the right one. The ECG can also aid in this diagnosis, as low voltage in limb leads is typical of amyloidosis despite a hypertrophic heart [50,51].

**Figure 6:**
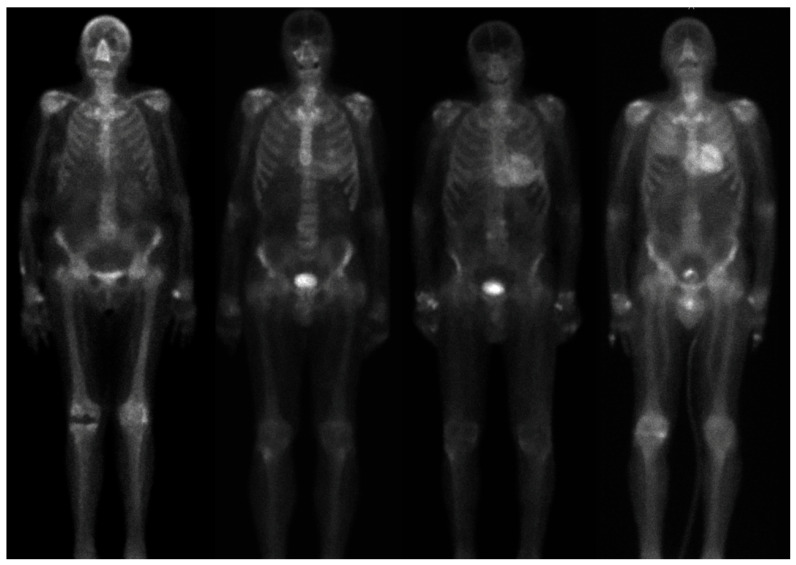
Representative examples of myocardial uptake of Tc-99m]HMDP, typical of aTTR cardiac amyloidosis. From left to right—no uptake (Perugini 0), mild uptake (Perugini 1), moderate uptake (Perugini 2), and high uptake (Perugini 3).

Scintigraphy with bisphosphonates (such as 99mTc-HMDP and DPD) or pyrophosphate (99mTc-PYP) has emerged as a crucial tool in the detection of cardiac amyloidosis, particularly for aTTR amyloidosis. This bone scintigraphy technique exploits the affinity of technetium-labeled bisphosphonates and pyrophosphates for amyloid deposits, allowing for the visualization of these deposits within the myocardium. This method has shown high sensitivity and specificity for imaging cardiac aTTR amyloid and is especially useful when traditional methods are inconclusive [17,52]. Consider the case of a 63-year-old cyclist who seeks medical attention for developing exercise dyspnea. An ECG reveals low QRS voltages in the limb leads and mild intraventricular conduction delay. An echocardiogram shows concentric hypertrophy with maximum ventricular wall thicknesses of 13–14 mm, a left ventricle of normal volume (despite the patient’s cycling activities), and grade II diastolic dysfunction. Blood tests reveal the absence of free light chains in both urine and plasma but show elevated troponin and BNP levels. For the suspicion of AaTR amyloidosis, a [Tc-99m]HMDP scintigraphy is performed, showing significant cardiac uptake (Perugini score 3) (Figure 6). Consequently, a biopsy of the periumbilical fat confirms the diagnosis. The patient is then directed towards specific therapy for aTTR cardiac amyloidosis with Tafamidis, a transthyretin stabilizer.

## 5. Future Directions and Innovative Techniques

### 5.1. Athlete with Distinctly Exercise-Induced Ventricular Arrhythmias

Evaluation of myocardial sympathetic innervation with MIBG scintigraphy may be useful for the evaluation of the arrhythmic risk in athletes. Cardiac denervation can promote arrhythmias by creating an imbalance in autonomic regulation, which affects the heart’s electrical stability. In heart failure settings, a reduction in cardiac sympathetic receptor densities have been correlated with increased arrhythmic risk [53]. Experimental models of arrhythmogenic cardiomyopathy have suggested the presence of cardiac denervation in areas affected by fibro-fatty replacement, which in turn exacerbates electrical instability and arrhythmogenesis [54]. Specifically, the regional sympathetic denervation of diseased myocardium leads to a supersensitivity to catecholamines, increasing the propensity for adrenergic-dependent ventricular arrhythmias. This denervation can cause a non-uniform refractory period in the myocardium, predisposing these regions to arrhythmias during sympathetic stimulation. In the context of evaluating athletes with adrenergic-dependent ventricular arrhythmias, whether in the presence of a substrate (such as non-ischemic LV scar) or in isolated forms, assessing cardiac innervation with MIBG SPECT could provide valuable pathophysiological insights and potentially aid in risk stratification.

### 5.2. Athlete with Non-Ischemic Myocardial Scar

The evaluation of athletes with non-ischemic left ventricular (LV) scar is a significant clinical challenge in sports cardiology. This condition has recently been recognized as a major cause of ventricular arrhythmias and SCD during sports [43,44]. Studies involving cardiac MRI in asymptomatic endurance athletes have shown that a notable minority (up to 17% in cohorts of male triathletes) exhibit non-ischemic late gadolinium enhancement (LGE) [55]. The presence of non-ischemic LV scar, typically detected via MRI after the finding of ECG abnormalities or exercise-induced arrhythmias, raises the issue of risk stratification to differentiate between benign (i.e., non-arrhythmogenic) LV scars and those with a potential for causing severe ventricular arrhythmias.

In addition to MIBG SPECT, a promising nuclear medicine technique is PET with FAPI (fibroblast activation protein inhibitor) labeled with 18F or 68Ga. This molecule binds to fibroblasts in areas of fibrosis, and especially when they are active. Initially used in oncology to study neoplastic lesions, recent studies have suggested potential applications in nuclear cardiology [56,57]. After a myocardial infarction, FAPI PET is positive only during the early phases of scar formation, with affinity diminishing as fibroblasts become inactive [58]. In non-ischemic scars, FAPI PET could theoretically distinguish between inactive, stable substrates (e.g., healed myocarditis scars) and active, potentially more arrhythmogenic scars (e.g., arrhythmogenic cardiomyopathy in development). This assessment could provide complementary information in the diagnostic and risk stratification process.

### 5.3. PET for Studying Mitochondrial Activity

Nuclear medicine techniques can evaluate mitochondrial activity, which is essential for energy production and cellular function, especially in athletes. PET tracers such as 18FFDG can assess metabolic activity and mitochondrial function by tracking glucose uptake and utilization [15]. Additionally, tracers such as 201Tl-chloride targeting Na/K-ATPase, although used mainly to detect myocardial ischemia, may be explored to better understand the ion transport and energy expenditure during intense physical activity [59]. These techniques can be potentially useful in studying conditions such as overtraining syndrome, where mitochondrial dysfunction may play a role. Understanding mitochondrial efficiency and detecting metabolic abnormalities can help optimize training regimens, prevent overtraining, and manage conditions such as mitochondrial myopathies that might affect athletic performance [60,61]. Finally, studies have shown that lactate metabolism and oxidative capacity are critical in understanding the cardiac adaptations and performance in athletes, and these metabolic processes can be evaluated using advanced PET imaging techniques [15].

## 6. Conclusions

Although nuclear cardiology is currently underused in the diagnostic work-up of athletes with suspected or diagnosed heart disease, there are specific scenarios where it may, or should, be employed. In fact, nuclear medicine techniques have the unique ability to evaluate inducible ischemia during maximal physical stress with high sensitivity. This is particularly important in cases of coronary artery anomalies where pharmacological stress might not replicate the physiopathological mechanism of ischemia. Furthermore, in cases of suspected sarcoidosis or amyloidosis, nuclear medicine can improve diagnostic accuracy compared to other imaging modalities, thereby facilitating a targeted biopsy. Additionally, in the future, nuclear medicine might provide relevant information for the arrhythmic risk stratification of athletes with arrhythmogenic conditions through the evaluation of sympathetic innervation, fibroblast activity, and mitochondrial function.

While the risks associated with ionizing radiation exposure must be considered, it is important to note that modern SPECT and PET equipment significantly reduce the required dose of radioactivity, making it comparable to or even lower than that of a CT scan. This advancement ensures that the benefits of accurate diagnosis and effective risk stratification outweigh the potential risks, particularly in the context of competitive sports, where precise evaluation is crucial.

While nuclear imaging techniques offer valuable diagnostic insights in the evaluation of athletes with suspected cardiac conditions, comprehensive data on their cost-effectiveness and relative risk in sports cardiology remain limited. Future research is needed to better quantify these aspects, which will be crucial for integrating these modalities into routine clinical practice and optimizing patient care in high-performance athletes.

## Figures and Tables

**Table 1:** Characteristics of radiotracers used in SPECT and PET.

Tracer	Physical Half Life	Uptake	First Pass Maximum Extraction Fraction, %
^82^Rubidium	73 s	Active, Na/K ATPase	65
^13^N-Ammonia	9.9 min	Enzimatic conversion to glutamate	82
[18F]Fluorodeoxyglucose	110 min	GLUT transporters	NA
[18F]Flurpiridaz	110 min	Passive; mitochondrial complex1	94
^201^Thallium	73 h	Active, Na/K ATPase	85
[99mTc]sestamibi	6 h	Passive; mitochondrial membrane potential	55–65
[99mTc]tetrofosmin	6 h	Passive; mitochondrial membrane potential	50–55
[99mTc]pyrophosphate	6 h	Extracellular calcium	NA
[123I]MIBG	13 h	Active	NA

NA: not analyze.

## Data Availability

Not applicable.
